# Eye contact modulates facial mimicry in 4-month-old infants: An EMG and fNIRS study

**DOI:** 10.1016/j.cortex.2018.05.002

**Published:** 2018-09

**Authors:** Carina C.J.M. de Klerk, Antonia F.de C. Hamilton, Victoria Southgate

**Affiliations:** a Centre for Brain and Cognitive Development, Birkbeck College, University of London, UK; b Institute of Cognitive Neuroscience, University College London, UK; c Department of Psychology, University of Copenhagen, Denmark

**Keywords:** Facial mimicry, Imitation, Infancy, Eye contact, EMG, fNIRS

## Abstract

Mimicry, the tendency to spontaneously and unconsciously copy others' behaviour, plays an important role in social interactions. It facilitates rapport between strangers, and is flexibly modulated by social signals, such as eye contact. However, little is known about the development of this phenomenon in infancy, and it is unknown whether mimicry is modulated by social signals from early in life. Here we addressed this question by presenting 4-month-old infants with videos of models performing facial actions (e.g., mouth opening, eyebrow raising) and hand actions (e.g., hand opening and closing, finger actions) accompanied by direct or averted gaze, while we measured their facial and hand muscle responses using electromyography to obtain an index of mimicry (Experiment 1). In Experiment 2 the infants observed the same stimuli while we used functional near-infrared spectroscopy to investigate the brain regions involved in modulating mimicry by eye contact. We found that 4-month-olds only showed evidence of mimicry when they observed facial actions accompanied by direct gaze. Experiment 2 suggests that this selective facial mimicry may have been associated with activation over posterior superior temporal sulcus. These findings provide the first demonstration of modulation of mimicry by social signals in young human infants, and suggest that mimicry plays an important role in social interactions from early in life.

## 1 Introduction

Humans' tendency to copy others' actions not only plays an important role in cultural learning (e.g., Legare and Nielsen, 2015, Tomasello et al., 1993) but also serves a highly social function (Over & Carpenter, 2013). Perhaps the clearest example of this can be seen when we spontaneously copy or ‘mimic’ others' behaviours (Chartrand & Bargh, 1999). Contrary to imitation, which is usually intentional and object- or effect-directed, mimicry is thought to occur outside of conscious awareness and is most common for non-object directed actions such as postures, gestures, and facial expressions (Chartrand & Bargh, 1999). This mimicry behaviour has been shown to play an important role in communication and affiliation (Chartrand & Lakin, 2013). For example, it influences liking and rapport between strangers, enhances the smoothness of social interactions (Chartrand & Bargh, 1999) and increases helpfulness (Van Baaren, Holland, Kawakami, & Van Knippenberg, 2004). Based on these findings it has been suggested that mimicry evolved to serve as ‘social glue’, binding individuals together and creating harmonious relationships, thereby facilitating survival (Lakin, Jefferis, Cheng, & Chartrand, 2003).

Studies on the neural basis of this phenomenon suggest that mimicry is supported by connections between brain regions involved in processing the kinematics of observed actions (e.g., superior temporal sulcus-STS), and regions that represent the motor commands needed to perform these actions (e.g., inferior frontal gyrus-IFG) (Likowski et al., 2012, Wang et al., 2011). As a result of this perception-action coupling, mimicry is often thought to be a pre-potent, automatic response tendency (e.g., Chartrand & Bargh, 1999). However, recent studies with adult participants have demonstrated that mimicry is flexibly modulated by social signals and social context. For example, it has been demonstrated that we have a stronger tendency to mimic others when they look at us (Bavelas, Black, Lemery, MacInnis, et al., 1986, Postma-Nilsenová et al., 2013, Wang et al., 2010), when we have been primed with self-related prosocial stimuli (Leighton et al., 2010, Wang and de C Hamilton, 2013), and when we have been given the goal to affiliate (Lakin & Chartrand, 2003). Together, these findings suggest that mimicry is a sophisticated mechanism that is flexibly employed depending on the social context and social goals, to enhance affiliation (Lakin et al., 2008, Wang and Hamilton, 2012).

Despite the important social functions that mimicry serves in adults, little is known about its development. Although recent studies have shown evidence of behavioural mimicry in early childhood (van Schaik & Hunnius, 2016) and mimicry of emotions in infancy (Geangu et al., 2010, Isomura and Nakano, 2016) and toddlerhood (Geangu, Quadrelli, Conte, Croci, & Turati, 2016), it is unknown whether the social modulation of mimicry, which is central to this phenomenon in adulthood, is also present early in life. The current study aimed to fill this gap by investigating whether mimicry is modulated by eye contact in 4-month-old infants.

Previous research suggests that eye contact is an important signal from very early in life; with infants demonstrating a preference for faces with open eyes (Batki, Baron-Cohen, Wheelwright, Connellan, & Ahluwalia, 2000), and faces that engage in mutual gaze (Farroni, Csibra, Simion, & Johnson, 2002) only a few days after birth. Furthermore, several studies have demonstrated that direct gaze influences the processing of concurrent or subsequent information from at least 4 months of age, a phenomenon that has been called the ‘eye contact effect’ (Senju & Johnson, 2009). For example, 4-month-old infants demonstrate better recognition memory for faces accompanied by direct compared to averted gaze (Farroni, Massaccesi, Menon, & Johnson, 2007), and a period of eye contact preceding a gaze shift seems to be a crucial prerequisite for gaze following in 4- and 6-month-olds (Farroni et al., 2003, Senju and Csibra, 2008).

In the present study we investigated the social modulation of mimicry by presenting 4-month-old infants with videos of models performing facial and hand actions accompanied by direct or averted gaze while we measured their facial and hand muscle responses using electromyography (EMG) (Experiment 1). We used EMG because it reveals sub-threshold muscle activity and likely provides a more objective, and more sensitive, measure of mimicry compared to that which is visible by eye. Based on the evidence for the ‘eye contact effect’ in 4-month-olds (Senju & Johnson, 2009), together with the previous research showing the modulation of mimicry by eye contact in adults (Wang et al., 2010), we hypothesized that infants would demonstrate greater mimicry of actions accompanied by direct gaze, compared to actions accompanied by averted gaze. In Experiment 2, the same group of infants observed the same videos of facial and hand actions accompanied by direct or averted gaze again while we used functional near-infrared spectroscopy (fNIRS) to investigate which brain regions may be involved in the modulation of mimicry by eye contact. Previous neuroimaging work with adult participants has demonstrated that STS plays a key role in processing gaze direction (e.g., Allison et al., 2000, Hoffman and Haxby, 2000), and that it is involved in the modulation of mimicry by eye contact in adult participants by modulating the sensory input to motor areas (Wang et al., 2011). Interestingly, a previous fNIRS study has demonstrated that 4-month-old infants also activate the posterior STS region when they observe direct gaze stimuli (Grossmann et al., 2008). Based on these studies, together with previous fMRI work that demonstrated that mimicry is supported by couplings between STS and IFG in adults (Likowski et al., 2012, Wang et al., 2011), we expected to find greater activation over STS, and consequently, IFG areas during the observation of actions accompanied by direct gaze.

## 2 Methods

### 2.1 Participants

A group of sixty 4-month-old infants participated in the study (*M* = 122 days; range 104–145 days; 30 girls). Out of these, 28 infants (*M* = 120 days; range 104–142 days; 11 girls) provided sufficient data to be included in the facial EMG analyses and 23 infants provided sufficient data to be included in the hand EMG analyses (*M* = 120 days; range 104–142 days; 8 girls) (Experiment 1), 31 infants (*M* = 120 days; range 104–141 days; 12 girls) provided sufficient data to be included in the NIRS analyses (Experiment 2). 18 infants contributed data to both the facial EMG and fNIRS analyses. See Supplementary materials for details on exclusion criteria. All included infants were born full-term, healthy and with normal birth weight. There were no differences in age or gender between the included and excluded infants, all *p*'*s* > .073. The study received approval from the institutional ethics committee, and written informed consent was obtained from the infant's caregiver prior to the start of the experiment.

## 3 Experiment 1: EMG

### 3.1 Procedure

**Fig. 1:**
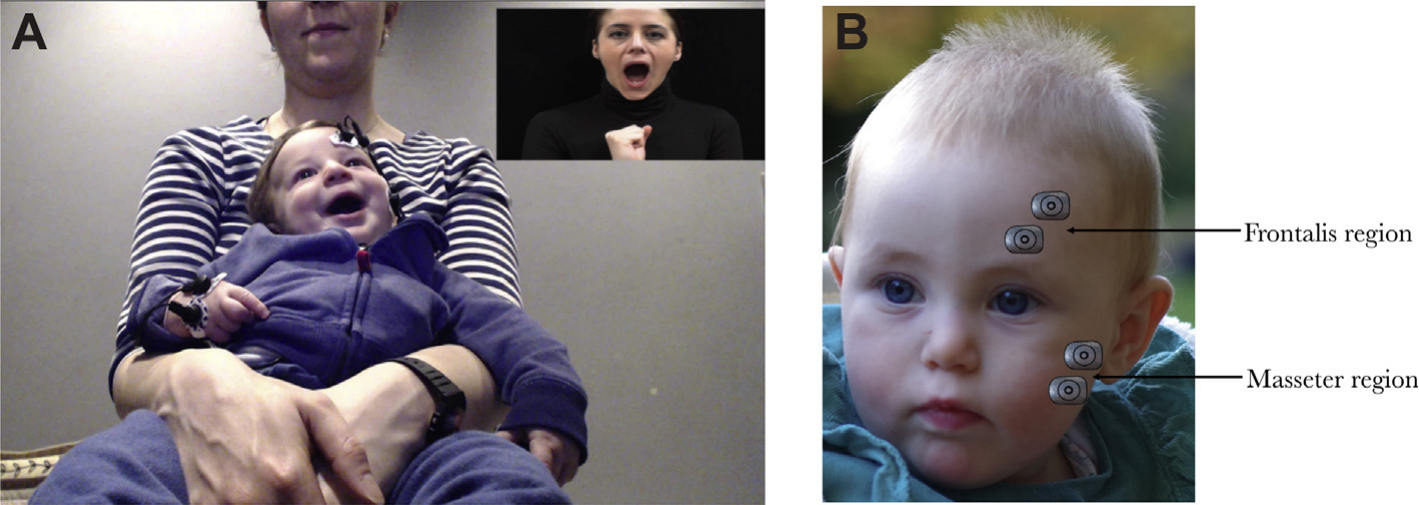
(A) Infant observing a Mouth_Direct trial. (B) Illustration of the facial EMG electrode placement.

**Fig. 2:**
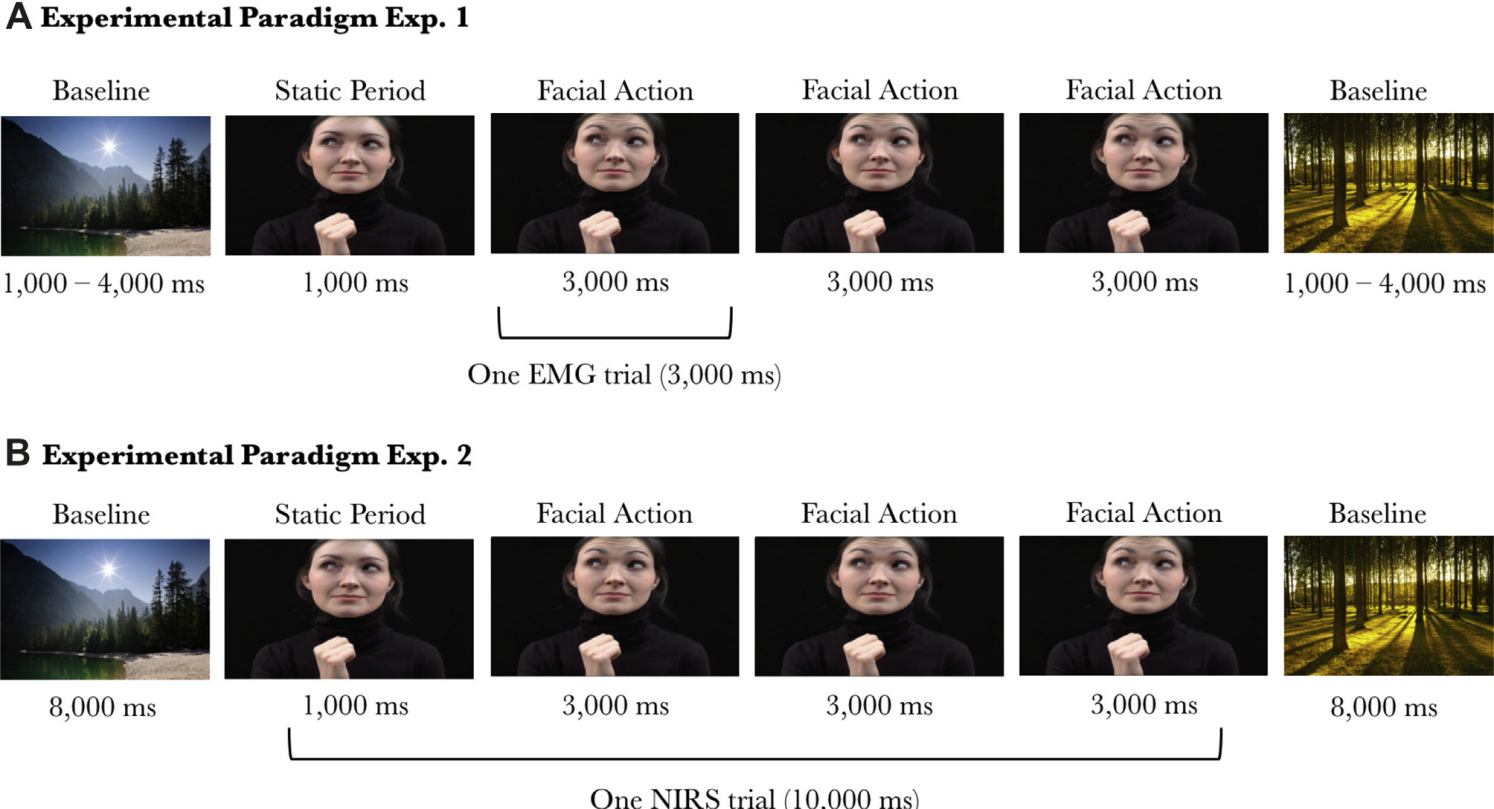
(A) Schematic overview of the stimulus presentation in Experiment 1. (B) Schematic overview of the stimulus presentation in Experiment 2.

The experiment took place in a dimly lit and sound attenuated room, with the infant sitting on their parent's lap at approximately 50–60 cm from a 58 cm screen (when viewed from 60 cm distance the stimuli subtended a visual angle of 27.2° × 46.0°). Infants were presented with videos of three female models performing eyebrow and mouth actions (i.e., eyebrow raising, frowning, tongue protrusion, and mouth opening) and hand actions (e.g., hand opening, and finger movements) accompanied by direct or averted gaze, while we measured activation over the eyebrow (frontalis) and mouth (masseter) region and the hand region using EMG (see Fig. 1a). There were six trial types: eyebrow actions accompanied by direct gaze (Eyebrow_Direct), mouth actions accompanied by direct gaze (Mouth_Direct), hand actions accompanied by direct gaze (Hand_Direct), eyebrow actions accompanied by averted gaze (Eyebrow_Averted), mouth actions accompanied by averted gaze (Mouth_Averted) and hand actions accompanied by averted gaze (Hand_Averted). We recorded twelve videos per model resulting in a total stimulus set of 36 different videos. Each video started with 1,000 ms during which the model did not perform any actions, followed by her performing three repeats of the same hand or facial action, each lasting 3,000 ms (See Fig. 2). Note that in hand trials the actress did not move her face, and in the face trials the hand was visible but stationary at the bottom of the screen. There were no gaze shifts in the averted gaze condition; the eyes were already averted at the onset of the video. The 10-sec videos were presented in a random order, alternated with Baseline trials consisting of static pictures of houses, animals, and landscapes with a random duration between 1,000 and 4,000 ms to allow for any mimicry responses to subside before the next video was presented (See Fig. 2a). If necessary, alerting sounds were played to draw the infant's attention back to the screen. Videos were presented until the infant had seen approximately 25 10-s Mimicry trials or until the infant's attention could no longer be attracted to the screen (mean number of presented trials = 26.5, SD = 2.8). Infants were video-recorded throughout the session.

### 3.2 EMG recording and processing

Bipolar EMG recordings were made using paediatric surface Ag/AgCl electrodes that were placed on the cheek, forehead, and hand with an inter-electrode spacing of approximately 1 cm. Surface EMG electrodes measure broad, non-selective firing of aggregates of motor units of muscle groups underlying and near the electrode sites (Lawrence & De Luca, 1983). Therefore, the electrodes on the cheek would mainly have picked up activity from the masseter muscle (involved in closing the mouth), but also from the underlying lateral pterygoid muscles (involved in opening the mouth), while the electrodes on the forehead recorded activity of the frontalis muscle (involved in raising the eyebrows) as well as the corrugtor supercilii (involved in frowning) (Fridlund & Cacioppo, 1986). Following Fridlund & Cacioppo's (1986) recommendation, we therefore use the terms ‘frontalis region’ and ‘masseter region’ to describe EMG activity measured over these areas (see Fig. 1b). The electrodes on the hand would have mainly picked up activity from the intrinsic hand muscles (e.g., the interossei muscles, and the lumbrical muscles). We use the term ‘hand region’ to describe EMG activity measured over this area. The electrodes were connected to Myon wireless transmitter boxes that amplified the electrical muscle activation, which was in turn recorded using ProEMG at a sampling rate of 2000 Hz. After recording, the EMG signal was filtered (high-pass: 30 Hz, low-pass: 500 Hz) smoothed (root mean square over 20 ms bins), and rectified (converted to absolute values).

Each 3,000 ms period during which a hand or facial action was performed by the model was treated as a separate trial (See Fig. 2a). Videos were coded offline and trials in which the infant did not see at least two thirds of the action were excluded from analysis. Additionally, facial action trials during which the infant vocalised, smiled, cried, or had something in their mouth (e.g., their hand or their clothing), and hand action trials during which the infant was moving their arms vigorously or holding onto something, were excluded from the analyses. Only infants with at least 3 trials per trial type were included in the analyses. On average, the included infants contributed 7.6 trials (SD = 3.0) per condition to the analyses; 6.9 in the Eyebrow_Direct condition (SD = 2.8), 8.5 trials in the Mouth_Direct condition (SD = 3.0), 7.1 trials in the Eyebrow_Averted condition (SD = 3.0), 7.6 trials in the Mouth_Averted condition (SD = 2.8), 7.7 trials in the Hand_Direct condition (SD = 3.3) and 7.3 trials in the Hand_Averted condition (SD = 3.5). The number of included trials did not differ between the Direct and Averted gaze condition, *p* = .719.

The EMG signal was segmented into 3,000 ms epochs, and the average activity in each epoch was normalised (i.e., expressed as z-scores) within each participant and each muscle group (masseter, frontalis, and hand region), before the epochs for each trial type were averaged together.^1^ This allows for meaningful comparison of values between muscle regions, as well as reducing the impact of individual differences in reactivity on the group mean.

As facial mimicry is defined as the increase in activation over corresponding muscles, in the absence of activation over non-corresponding muscles during the observation of facial actions (e.g., McIntosh et al., 2006, Oberman et al., 2009), we calculated a facial mimicry score per trial by subtracting EMG activity over the non-corresponding muscle region from EMG activity over the corresponding muscle region (e.g., on an eyebrow trial we subtracted activity over the masseter region from activity over the frontalis region, so that a more positive score indicates more facial mimicry).

## 4 Results

### 4.1 Facial mimicry

**Fig. 3:**
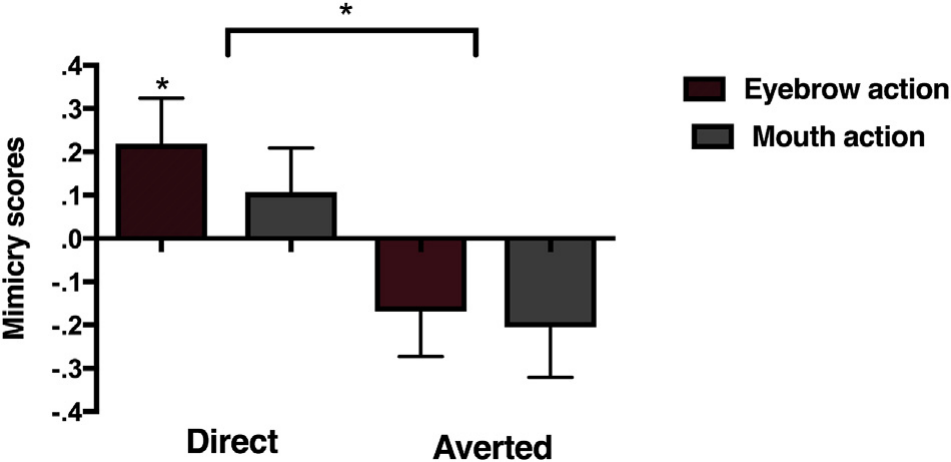
Mean Mimicry scores (activation over the corresponding muscle region minus activation over the non-corresponding muscle region) during the observation of eyebrow and mouth actions in the Direct and Averted gaze condition. *p < .05. Error bars indicate 1 SEM.

A repeated measures analysis on the Mimicry scores (i.e., activation over the corresponding muscle region minus activation over the non-corresponding muscle region) with Gaze direction (Direct *vs* Averted) and Action type (Eyebrow *vs* Mouth) demonstrated a significant main effect of Gaze direction, *F* (1, 27) = 7.997, *p* = .009, *η*_*p*_^*2*^ = .229 and a significant main effect of Action type, *F* (1, 27) = 4.690, *p* = .039, *η*_*p*_^*2*^ = .148. There was no interaction between Gaze direction and Action type, *F* (1, 27) = .040, *p* = .736, *η*_*p*_^*2*^ = .004. The main effect of Action type was driven by the Mimicry scores being greater for the eyebrow action trials than the mouth action trials. As can be seen in Fig. 3 there was significantly greater mimicry in the Direct gaze compared to the Averted gaze condition. Follow-up one sample *t*-tests demonstrated that the mimicry scores in the Direct Gaze eyebrow action condition were significantly different from zero, *t*(27) = 2.085, *p* = .047. Thus, we found greater mimicry of facial actions accompanied by direct compared to averted gaze, and this effect seemed strongest for the observation of eyebrow actions (See supplementary materials for additional analyses on the z-scored EMG activity per muscle region).

### 4.2 Hand mimicry

**Fig. 4:**
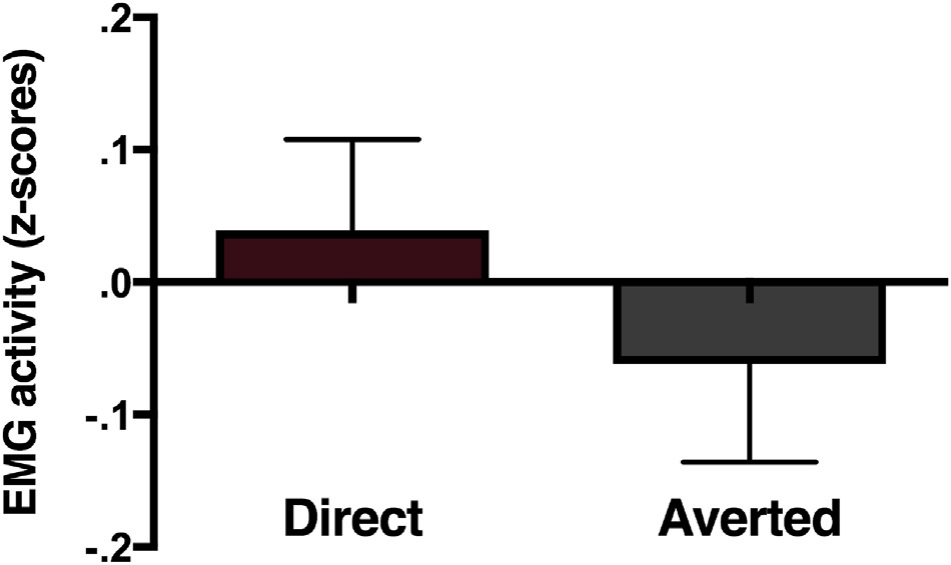
Mean EMG-activity (z-scores) over the hand region during the observation of hand actions accompanied by direct and averted gaze. Error bars indicate 1 SEM.

A repeated measures analysis on the EMG activity over the hand region during the observation of hand actions with Gaze direction (Direct *vs* Averted) as within subject factors demonstrated no significant effect of gaze direction, *F* (1, 22) = .507, *p* = .484, *η*_*p*_^2^ = .023 (see Fig. 4). Additionally, the EMG activity over the hand area was not significantly different from zero in either condition. Thus, we did not find evidence for mimicry of hand actions, nor of any effect of condition over the hand areas.

## 5 Experiment 2: fNIRS

After Experiment 1, infants had a nap to make sure they were refreshed before participating in Experiment 2. Experiment 2 aimed to investigate which brain regions may be involved in modulating facial mimicry by eye contact using fNIRS. As we did not find evidence for mimicry of hand actions in Experiment 1, Experiment 2 focussed on the neural responses during the observation of facial actions accompanied by direct and averted gaze (see Supplementary materials for the neural responses to the hand action condition). Infants observed the same stimuli as in Experiment 1, with the only difference being that the baseline stimuli had a duration of 8 s to allow the haemodynamic response to return to baseline levels (see Fig. 2b). The experiment took place in a dimly lit and sound attenuated room, with the infant sitting on their parent's lap at approximately 90 cm from a 117 cm plasma screen (when viewed from this distance the stimuli subtended a visual angle 27.5° × 51.3°).

Videos were presented until the infant had seen approximately 20 10-sec Mimicry trials or until the infant's attention could no longer be attracted to the screen (mean number of presented trials = 21.8, SD = 4.1).

### 5.1 fNIRS recording and processing

fNIRS data was recorded using the UCL-NIRS topography system, which uses two continuous wavelengths of near-infrared light (770 and 850 nm) to detect changes in oxyhemoglobin (HbO_2_) and deoxyhemoglobin (HHb) concentrations in the brain (Everdell et al., 2005). Infants wore a custom-built headgear with sources and detectors embedded within a left and right hemisphere array, resulting in a total of 26 channels with a source-detector separation of 20 mm. Based on the understanding of the transportation of near-infrared light through tissue, this source-detector separation was predicted to penetrate up to a depth of approximately 10 mm from the skin surface, allowing measurement of both the gyri and parts of the sulci near the surface of the cortex (Lloyd-Fox, Blasi, & Elwell, 2010). A mark indicating the midpoint of the headgear was aligned to the infant's nasion. Additionally, for the sides of the headgear the midpoint of the lower row of channels was aligned with the pre-auricular points on the average 4-month-old (T3 on the left hemisphere and T4 on the right hemisphere in the 10–20 system) (Lloyd-Fox et al., 2009). Previous research using co-registration of fNIRS and MRI recordings using the same headgear has demonstrated that it allows measurement of haemodynamic responses in cortical regions corresponding to IFG, STS, and TPJ areas (Lloyd-Fox et al., 2014).

Videos were coded offline and trials in which the infant did not attend to at least 2 of the 3 facial actions, or trials during which the infant was crying were excluded from analyses. We also excluded baseline trials during which the infant was looking at their parents' face or their own limbs in movement. Note that while facial mimicry as measured by EMG can be recorded on a millisecond scale, the haemodynamic response takes several seconds to build up. Therefore, while in Experiment 1 we treated each 3,000 ms period during which a facial action was performed by the model in the video as a separate trial, for the fNIRS analyses in Experiment 2 we treated the 10,000 ms videos including three repeats of the same facial action as one trial (see also Fig. 2b). Only infants with at least 3 trials per experimental condition (Face_Direct, Face_Averted^2^) were included in the analyses. On average, the included infants contributed 5.8 trials per condition to the analyses; 5.9 trials in the Face_Direct condition, and 5.7 in the Face_Averted condition. The number of included trials did not significantly differ between the Direct and Averted gaze condition, *p* = .45.

The data were converted to.nirs format and channels were excluded if the magnitude of the signal was greater than 97% or smaller than 3% of the total range for longer than 5 s during the recording. The data were pre-processed using HOMER2, a Matlab software package (MGH-Martinos Center for Biomedical Imaging, Boston, MA, USA; Huppert, Diamond, Franceschini, & Boas, 2009). Channels with raw intensities smaller than .001 or bigger than 10 were excluded, and motion artefacts were corrected using wavelet analyses with an interquartile range of .5. Hereafter the data were band-pass filtered (high-pass: .01 Hz, low-pass: .80 Hz) to attenuate slow drifts and high frequency noise. Infants for whom more than 30% of channels were excluded due to excessive artefacts were excluded from analysis. We also excluded any channels that did not yield clean data for at least 70% of the infants from the analysis. This resulted in the exclusion of 2 channels (channels 1 and 5). The data were converted to relative concentrations of oxygenated (HbO_2)_ and deoxygenated haemoglobin (HHb) using the modified Beer–Lambert law (path length factor: 5.1; Duncan et al., 1996). Relative changes in HbO_2_ and HHb, were computed for 17-sec long epochs starting 2 s before the onset of each trial and ending 5 s after trial offset. The 2-sec pre-experimental window was considered as a baseline, and the mean HbO_2_ and HHb concentrations during this period were subtracted from the concentrations in the 15-sec analysis period. The signals were then averaged across trials for each channel and condition.

We adopted a similar approach to Lloyd-Fox, Széplaki-Köllőd, Yin, and Csibra (2015): first we quantified the mean haemodynamic concentration changes during five 3-sec sub-epochs following trial onset. Hereafter we performed repeated measures analyses with the 5 time bins and the two conditions (Face_Direct *vs* Face_Averted) as within subjects factors to identify channels for which there was a significant HbO_2_ increase or a significant HHb decrease from baseline when both conditions were considered together (as evidenced by a significant main effect of time). Repeated-measures analyses were then conducted on each of these pre-selected channels to assess whether there were differences in the haemodynamic response between the two conditions (Face_Direct *vs* Face_Averted). To ensure statistical reliability, we considered that activation at a single channel would be reliable only if it was accompanied by significant activation at an adjacent channel (Lloyd-Fox, Blasi, Everdell, Elwell, & Johnson, 2011). Hereafter we investigated the relationship between those channels that were sensitive to gaze direction and the facial mimicry effects measured in Experiment 1, to identify the brain regions that may play a role in modulating facial mimicry by eye contact.

## 6 Results

The initial analyses identified 12 channels that showed a significant haemodynamic response, i.e., an increase in HbO_2_ and/or a decrease in HHb during the trial period compared to the baseline period (see Supplementary Table 1). For two of these channels we found a significantly greater HbO_2_ response to the Face_Direct compared to the Face_Averted condition (channel 10: main effect of condition, *F*(1,30) = 5.279, *p* = .029, indicating a significantly greater HbO_2_ response to the Face_Direct condition throughout the analysis period; channel 9: interaction between time and condition *F*(4,120) = 2.535, *p* = .044, indicating a significantly greater increase in the HbO_2_ response to the Face_Direct condition over the analysis period). Monte-Carlo simulations for our array revealed that a per-channel significance threshold of *p* < .044 corresponds to a whole-array threshold of *p* < .054 for finding two adjacent channels activated by chance.

**Fig. 5:**
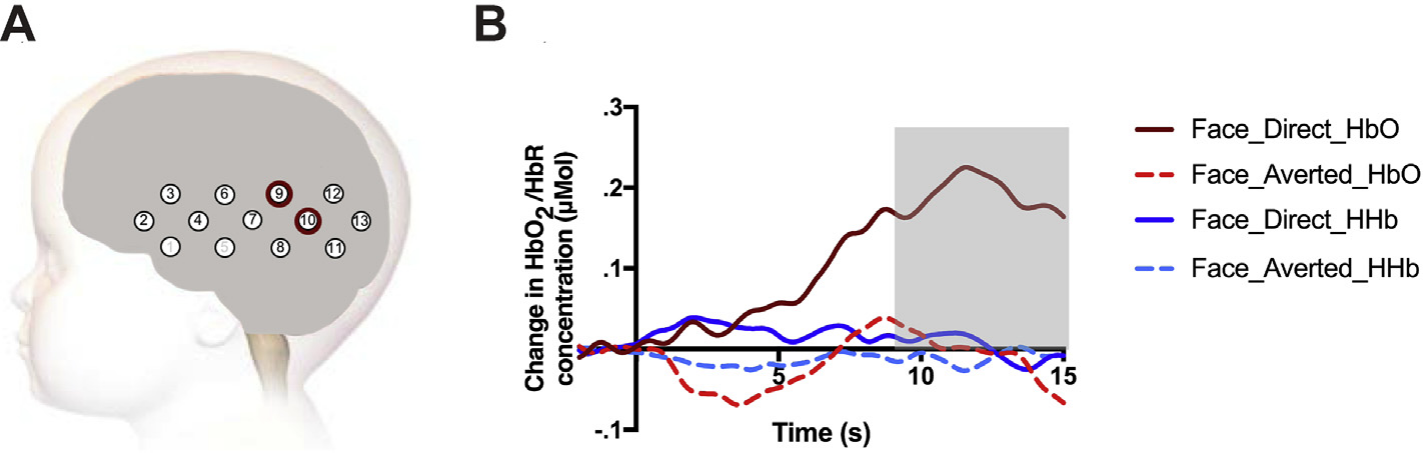
(A) The location of the fNIRS channels with significant increases in HbO_2_ for the Face_Direct compared to the Face_Averted condition. Grey numbers indicate excluded channels (1 and 5). (B) Time course of the grand averaged haemodynamic responses over the same two channels for both conditions. The grey area indicates the interval where the difference in the HbO_2_ response between the two conditions reached significance.

Using a standardized scalp surface map of the fNIRS channel coordinates for this array and this age range (Lloyd-Fox et al., 2014) we identified the location of these channels as overlying the left posterior STS region (see Fig. 5a). We averaged the HbO_2_ responses over these two channels together to investigate the time course of this effect. We found a greater increase in HbO_2_ to the Face_Direct condition relative to the Face_Averted condition over pSTS over the last two time windows: 9–12 sec post-stimulus onset: *t*(30) = 2.164, *p* = .039; and 12–15 s post-stimulus onset: *t*(30) = 2.257, *p* = .031. These effects are depicted in Fig. 5b. There were no channels that showed a greater haemodynamic response to the Face_Averted compared to Face_Direct condition, and there were also no significant effects of condition for the HHb signal.

The HbO_2_ responses over pSTS and IFG channels were significantly correlated, e.g., the differential HbO_2_ response to the Face_Direct compared to the Face_Averted condition over channel 10 (pSTS) was correlated with the differential HbO_2_ response to the Face_Direct compared to the Face_Averted condition over channel 4 (IFG) at 9–12 sec post-stimulus onset, *r* (24) = .457, *p* = .025. Follow-up analyses demonstrated a significant positive relationship between HbO_2_ responses over channel 10 (pSTS) and channel 4 (IFG) in the Face_Direct condition, *r* (24) = .537, *p* = .007, and no relationship between HbO_2_ responses over channel 10 (pSTS) and channel 4 (IFG) in the Face_Averted condition, *r* (24) = .035, *p* = .871. Thus indicating that the relationship between activation over pSTS and IFG channels was only present in the Face_Direct condition. Nevertheless, we did not find significant differences between the Face_Direct and Face_Averted condition over channels overlying IFG areas.

### 6.1 Relationship between EMG and fNIRS data

**Fig. 6:**
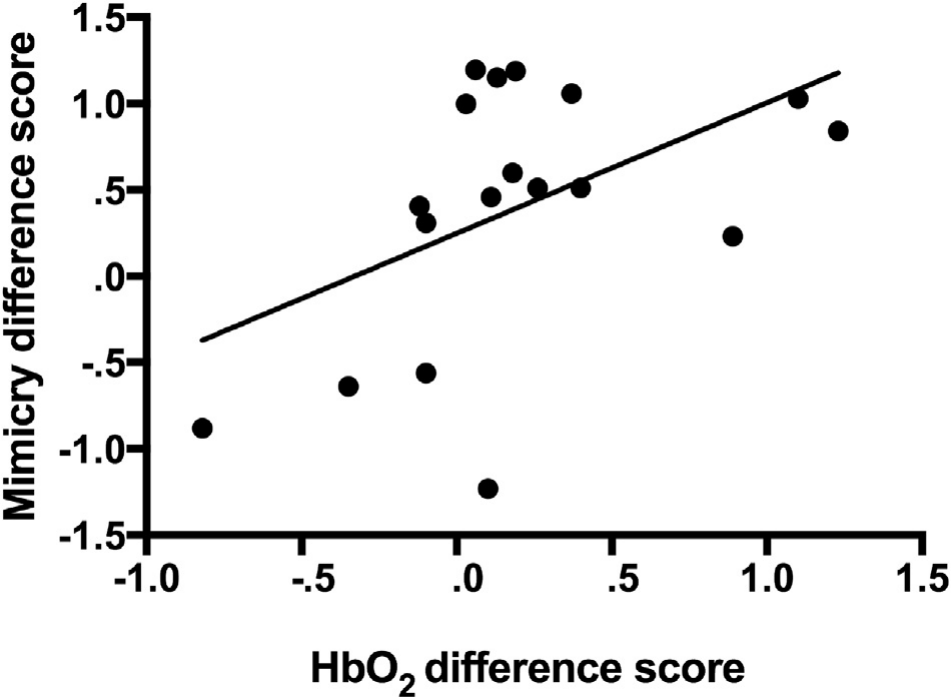
Relationship between the HbO_2_ difference score (HbO_2_ in the Face_Direct condition minus HbO_2_ in the Face_Averted condition as measured in Experiment 2) and the Mimicry difference score (average facial mimicry score in the Direct gaze condition minus the average facial mimicry score in the Averted gaze condition as measured in Experiment 1).

Hereafter we investigated the relationship between the haemodynamic response over the channels overlying left pSTS in Experiment 2 and infants' facial mimicry responses in Experiment 1. To limit the number of correlational analyses to be ran, we calculated a Mimicry difference score (infants' average facial mimicry score in the Direct gaze condition minus their average facial mimicry score in the Averted gaze condition) and a HbO_2_ difference score (the average HbO_2_ response over pSTS in the Face_Direct condition minus the average HbO_2_ response over pSTS in the Face_Averted condition). The differential HbO_2_ response over left pSTS (channels 9 and 10) in the 9–12 sec time window was significantly correlated with the differential mimicry score, *r*(16) = .496, *p* = .036 (lower 95% CI = .101, upper 95% CI = .766; estimated using bootstrapping with 1,000 replication samples). Thus, infants who showed a greater HbO_2_ response over left pSTS areas when observing facial actions accompanied by direct gaze compared to averted gaze (at 9–12 sec post-stimulus onset) also showed greater mimicry of facial actions accompanied by direct gaze (see Fig. 6). We did not find any relationship between the haemodynamic responses over IFG channels and the mimicry scores.

## 7 Discussion

In this study we adopted a novel approach by measuring 4-month-old infants' muscle responses using EMG and their haemodynamic responses using fNIRS to investigate a) the presence of mimicry early in infancy, b) whether this early mimicry is modulated by gaze direction, as it is in adults, and c) the neural mechanisms underlying the modulation of mimicry. We demonstrated that direct gaze cues enhance mimicry of facial actions (in particular eyebrow actions) in 4-month-old infants (Experiment 1), and that this modulation of mimicry seems to be accompanied by activation over left pSTS areas (Experiment 2). Together with recent work by Isomura and Nakano (2016)–that showed evidence for mimicry of emotional facial expressions in 5-month-olds–these findings demonstrate that spontaneous facial mimicry is an early emerging phenomenon that is present from at least 4-months of age. Importantly, the current results show that mimicry is already influenced by social signals at 4-months of age, suggesting that the foundations for the affiliative role that mimicry plays in social interactions are present from early in life.

Before we discuss the interpretation of our findings there are several things to note about the EMG results. Firstly, only the mimicry of eyebrow actions was significantly different from zero. One possible explanation for this finding might be that if eye contact is crucial for eliciting mimicry behaviour in young infants, then the facial actions involving the eye region may have been more effective in this respect. Alternatively, the data over the mouth region may have been noisier because of sub-threshold muscle activation unrelated to the stimulus presentation, such as swallowing. However, note that we did not find any significant differences between mimicry of mouth and eyebrow actions accompanied by direct gaze, thus although the eyebrow mimicry may have been stronger, it was not systematically different from the mouth mimicry in this condition. A second thing to note is that we seem to find counter-mimicry effects in the Averted gaze condition. This effect likely results from the fact that the data were converted to z-scores. If the EMG activity in the direct gaze condition was consistently higher than the mean, then the EMG activity in the averted gaze condition would inevitably have been lower than the mean. Converting the data to z-scores is standard practise in EMG analyses and allows for meaningful comparisons between muscle regions to be made. Although it is in principle possible that the effect of gaze direction was driven by counter-mimicry in the averted gaze condition, there is no previous theoretical or empirical work that would support this interpretation, and crucially the mimicry scores in the averted gaze condition were not significantly different from zero. Thus we believe that our results are most consistent with the interpretation that we found mimicry of facial actions accompanied by direct gaze but not for facial actions accompanied by averted gaze. Finally, we did not find evidence for mimicry of hand actions, nor of any effect of condition over the hand areas. It has been suggested that the perceptual-motor couplings that support mimicry develop through associative learning during correlated sensorimotor experience (Catmur, Walsh, & Heyes, 2009), and recent studies with adults (reviewed in Cook, Bird, Catmur, Press, & Heyes, 2014) and infants (de Klerk, Johnson, Heyes, & Southgate, 2015) have provided support for this idea. For hand actions, the necessary sensorimotor input for the formation of these couplings likely comes from infants' tendency to observe their own hands (Del Giudice, Manera, & Keysers, 2009). Therefore, if there are large individual differences in infants' relative interest in their own hands we would also expect to find large variability in the hand mimicry responses, which may explain the absence of hand mimicry in this study. Future research should investigate this possibility. With respect to the absence of an effect of eye contact on the hand mimicry, one possibility is that not all infants noticed the gaze direction while focussing on the hand actions in the lower part of the screen paediatric.

Several lower-level and higher-level mechanisms may play a role in the modulation of facial mimicry by eye contact, including associative learning, spatial attention, arousal, and social communication. We will discuss each of these in turn. Firstly, for facial actions, the necessary sensorimotor input for the formation of perceptual-motor couplings that support mimicry is thought to come from parents' tendency to copy their infant's facial actions (Ray & Heyes, 2011). As these imitative interactions would typically be accompanied by mutual gaze between the infant and the parent, it is possible that the facial actions accompanied by direct gaze were more effective in activating the associated corresponding motor representations because of the greater context similarity. Thus, one possibility is that our findings were the result of context effects in associative learning (Cook, Dickinson, & Heyes, 2012).

Another possibility is that the averted gaze served as a spatial cue leading infants' attention away from the face (Friesen, Moore, & Kingstone, 2005). However, the models in our videos did not perform any gaze shifts (the eyes were already averted at the onset of the video) and previous research suggests that perceived motion is a necessary factor for gaze cuing in 4-month-old infants (Farroni, Johnson, Brockbank, & Simion, 2000). Additionally, the eye tracking data that was recorded from a subset of the infants (see Supplementary materials) demonstrated that there was actually *greater* overt attention to the face in the averted gaze condition, making it unlikely that the absence of mimicry in this condition was driven by a lack of attention to the stimuli. Nevertheless, previous research suggests that averted gaze cues can lead to reduced processing of facial information (Farroni et al., 2007), and therefore it remains possible that eye contact is a necessary prerequisite for the effective processing of facial features and actions.

Finally, having a stranger gaze directly at oneself has been shown to increase autonomic arousal in adults (Nichols & Champness, 1971). Thus the observation of the facial actions accompanied by direct gaze may have been associated with increased general arousal. This could in turn have led to increased encoding of the stimuli and greater activation of the associated motor representations, a process termed *input modulation* (Heyes, 2013). We did not find increased overt attention to the faces with direct gaze (see Supplementary materials), which makes this interpretation seem less likely. However, looking is not always equivalent to attending (Aslin, 2012, Lansink and Richards, 1997) and therefore we cannot completely rule out the possibility that there may have been better encoding of the facial actions accompanied by direct gaze.

There are also higher-level mechanisms that have been put forth to explain the social modulation of mimicry behaviours in adults. First of all, it has been suggested that direct gaze serves as an important *ostensive cue*, signalling the intent to communicate with the perceiver, leading to increased social learning (Csibra & Gergely, 2009). Previous work has demonstrated that eye contact indeed modulates imitative responses in typically developing infants and toddlers (e.g., Carpenter et al., 1995, Király et al., 2013; Vivanti & Dissanayake, 2014). For example, Király et al. (2013) found that eye contact during the demonstration of an unusual, novel action was critical to elicit copying behaviour in 14-month-old infants. Thus, it is possible that direct gaze accompanying the facial actions in the current study may have communicated to the infants that these actions were demonstrated *for them*, increasing their social relevance and resulting in a greater tendency to copy. Secondly, mimicry, and in particular facial mimicry, has been suggested to be an important form of implicit *non-verbal communication* that can convey messages like ‘I feel your pain’ or, more generally, ‘I am like you’ to the observer (Bavelas, 2007, Bavelas, Black, Lemery, & Mullett, 1986). According to this account, eye contact might enhance mimicry responses because it allows the mimicker to communicate liking and rapport to the mimickee. In line with this idea, there is evidence that some imitative responses in every day interactions between toddlers serve communicative-affiliative functions (Eckerman et al., 1989, Nadel, 2002). As far as we are aware the social communicative function of mimicry has not been directly investigated in younger infants. However, previous research has shown that infants play an active role in face-to-face communication from early in life, and start to coordinate their facial expressions with the presence of eye contact with the parent between 3 and 6 months of age (Kaye and Fogel, 1980, Yale et al., 2003). Thus, if mimicry indeed serves as an important means for communication without language, it seems plausible that preverbal infants would show more mimicry behaviour when they are in a communicative setting, e.g., in an interaction with someone who is looking at them.

These low- and high-level hypotheses are not mutually exclusive, and several of the mechanisms described above may have played a role in modulating infants' facial mimicry responses in the present study. In fact, the lower-level mechanisms may describe the possible processes through which the social modulation of mimicry driven by higher-level motivations takes place. For example, the infant's desire to communicate may lead to greater arousal and attention whenever a communicative signal such as direct gaze is perceived, and this heightened attention to their interaction partner may in turn lead to an increase in mimicry behaviour. Future studies should investigate the relative importance of these different mechanisms, for example by measuring pupil dilation or skin conductance alongside EMG responses to look at the role of arousal, or by relating infants' previous sensorimotor experience with facial actions to their mimicry responses to shed light on the role of associative mechanisms.

Analysis of infants' haemodynamic responses (Experiment 2) showed that pSTS was sensitive to the gaze direction accompanying the facial actions. Replicating previous findings (Grossmann et al., 2008), this region showed a significantly greater haemodynamic response in the direct gaze compared to the averted gaze condition. Given STS' known role in processing gaze direction (Allison et al., 2000, Hoffman and Haxby, 2000), it is unclear based on this finding alone whether the greater haemodynamic response over left pSTS was driven by processing of the direct gaze by itself or by processing of the facial actions *accompanied* by direct gaze. However, importantly, infants who showed a greater HbO_2_ response over left pSTS when observing facial actions accompanied by direct gaze compared to averted gaze in Experiment 2 also showed greater mimicry of facial actions accompanied by direct gaze compared to averted gaze in Experiment 1. These findings are consistent with previous adult studies that have demonstrated that STS plays a role in the modulation of mimicry by eye contact (Wang & Hamilton, 2012). In adults, this modulation is thought to be supported by mPFC exerting a top-down influence on STS, thereby modulating the sensory input to motor areas (Wang and Hamilton, 2012, Wang et al., 2011). Together with previous findings that both mPFC and STS are activated when 4-month-old infants observe direct gaze cues (Grossmann et al., 2008), the current results could be taken to suggest that there may already be some form of functional connectivity between these areas. However, an obvious limitation of the current work is that we did not measure activation over mPFC. Additionally, although we found significant correlations between HbO_2_ responses over pSTS and IFG channels (in particular in the Face_Direct condition), the condition difference over IFG channels, and the relationship between HbO_2_ responses over IFG channels and the Mimicry scores, did not reach significance. Possibly this was due to greater noisiness of the data over this area, as two of the channels over the left anterior temporal cortex were excluded from analyses because they did not yield clean data for at least 70% of the infants (see fNIRS recording and processing section). Nevertheless, based on these findings it is unclear whether mimicry in infancy is supported by links between STS and IFG, as it is in adulthood. Finally, we recorded the EMG and NIRS activity in separate sessions, which increases the uncertainty about whether the neural responses measured in Experiment 2 indeed reflected the neural responses supporting mimicry behaviour. Future studies should measure functional brain responses, and ideally functional connectivity, simultaneously with mimicry behaviours to investigate: 1) the relationship between the neuronal activation and mimicry responses on a trial-by-trial basis, and 2) mPFC's role in orchestrating activation in STS and IFG, as well as the connections between these latter two regions as myelination of the relevant long-range connections increases over the course of development (Johnson, Grossmann, & Kadosh, 2009).

Although our study establishes that mimicry is present and modulated by social context by 4 months, we do not know whether the mimicry that we describe here is the same phenomenon that has been described in newborn infants. While the existence of newborn imitation has been the topic of much debate and scepticism (e.g., Jones, 2009, Ray and Heyes, 2011), EMG may provide a powerful way to move that debate forward. For example, if spontaneous facial mimicry objectively measured by EMG were also modulated by social context in neonates, it would provide compelling evidence against the argument that neonatal imitation is simply a reflexive behaviour (Anisfeld, 1991).

## 8 Conclusion

The current study demonstrates that direct gaze is a powerful social signal that enhances facial mimicry in 4-month-old infants, and that this modulation is associated with activation over left pSTS. These findings provide the first demonstration of modulation of mimicry by social signals in young human infants, and suggest that mimicry may play an important role in social interactions from early in life.

## Footnotes

1 Preliminary time course analyses on the baseline-corrected EMG signal indicated that for those infants who showed a mimicry response, the activation over the corresponding muscles persisted for the full duration of the observed action (see Supplementary Fig. 1). These analyses also demonstrated that the mimicry responses sometimes continued into the next baseline (see Supplementary Fig. 1b). Therefore, we decided to compare standardized activation over the different muscle groups instead of using baseline-corrected values.

2 We did not split the trials into four categories because not all infants had at least 3 good trials for the four different trial types. Instead we collapsed the Mouth and Eyebrow trials into the Face_Direct and Face_Averted condition.
