# Supplementary material for: Eye contact modulates facial mimicry in 4-month-old infants: An EMG and fNIRS study
Source: Cortex. 2018 Sep;106:93–103. doi: 10.1016/j.cortex.2018.05.002 (PMC6143479; doi:10.1016/j.cortex.2018.05.002)
Supplement: Multimedia component 1 [file mmc1.docx]

**Supplementary materials**

**Participants exclusions**

32 infants were excluded from the facial EMG analyses due to technical error (N = 3), or because they did not provide enough trials for analyses due to: fussiness (N=10), inattentiveness (N =12), because they constantly vocalised or repeatedly put their fingers in their mouth (N = 5), or because they had raised eyebrows throughout the experiment (N =2). An additional five infants were excluded from the hand EMG analyses due to: technical error (lost EMG signal from the hand EMG transmitter box) (N=1), because they were holding on to their mother’s hands for the entire session (N=3), or because their hands were not visible in the video recording for a sufficient number of trials (N=1).

29 infants were excluded from the NIRS analyses due to: positioning of the headgear (headgear shifted too far up, N = 3), too many bad channels because of thick dark hair (N=4) or excessive movement artefacts (N = 2), or because they did not provide the minimum of 3 good trials per condition due to: fussiness (N =8) or inattentiveness (N=12).

**
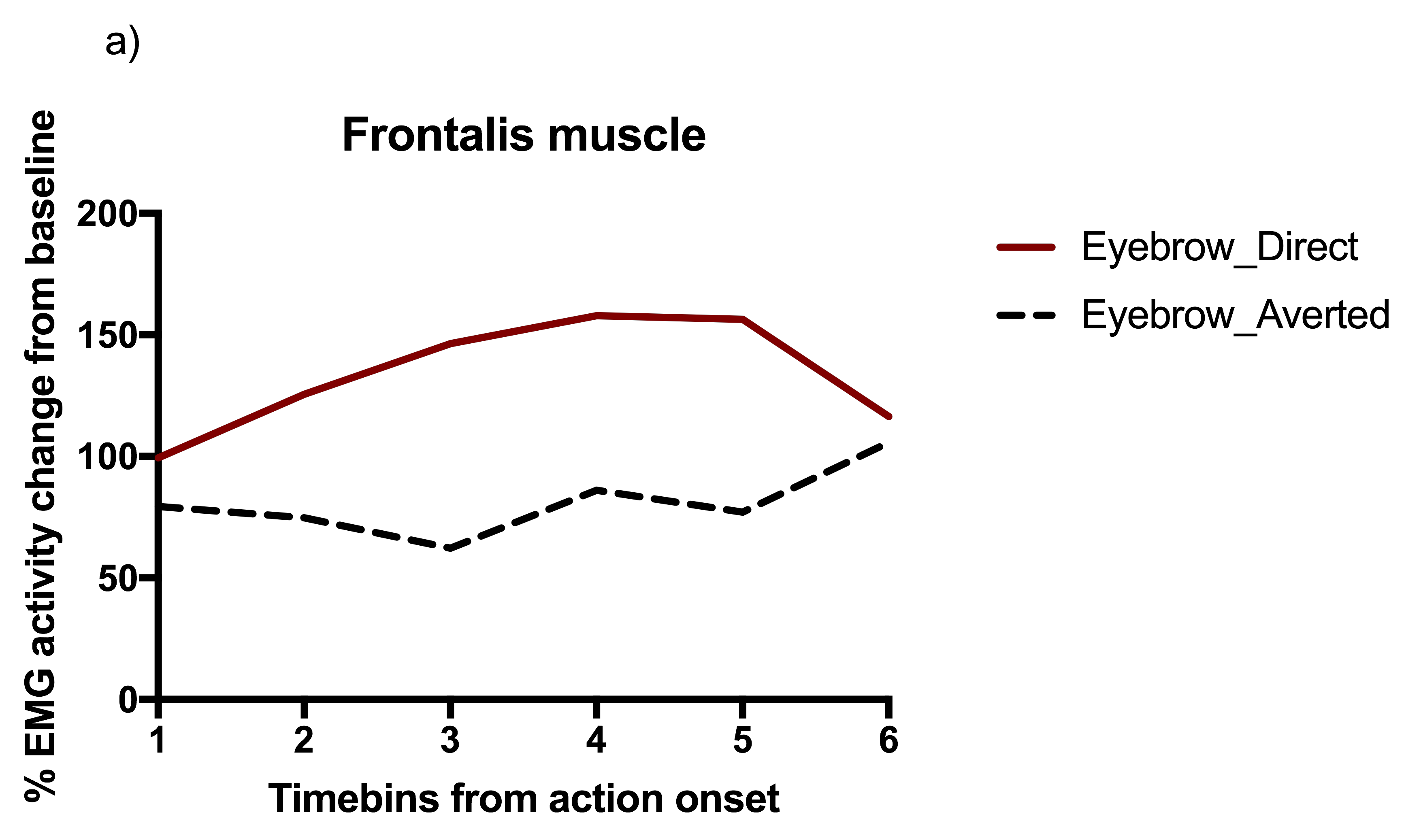

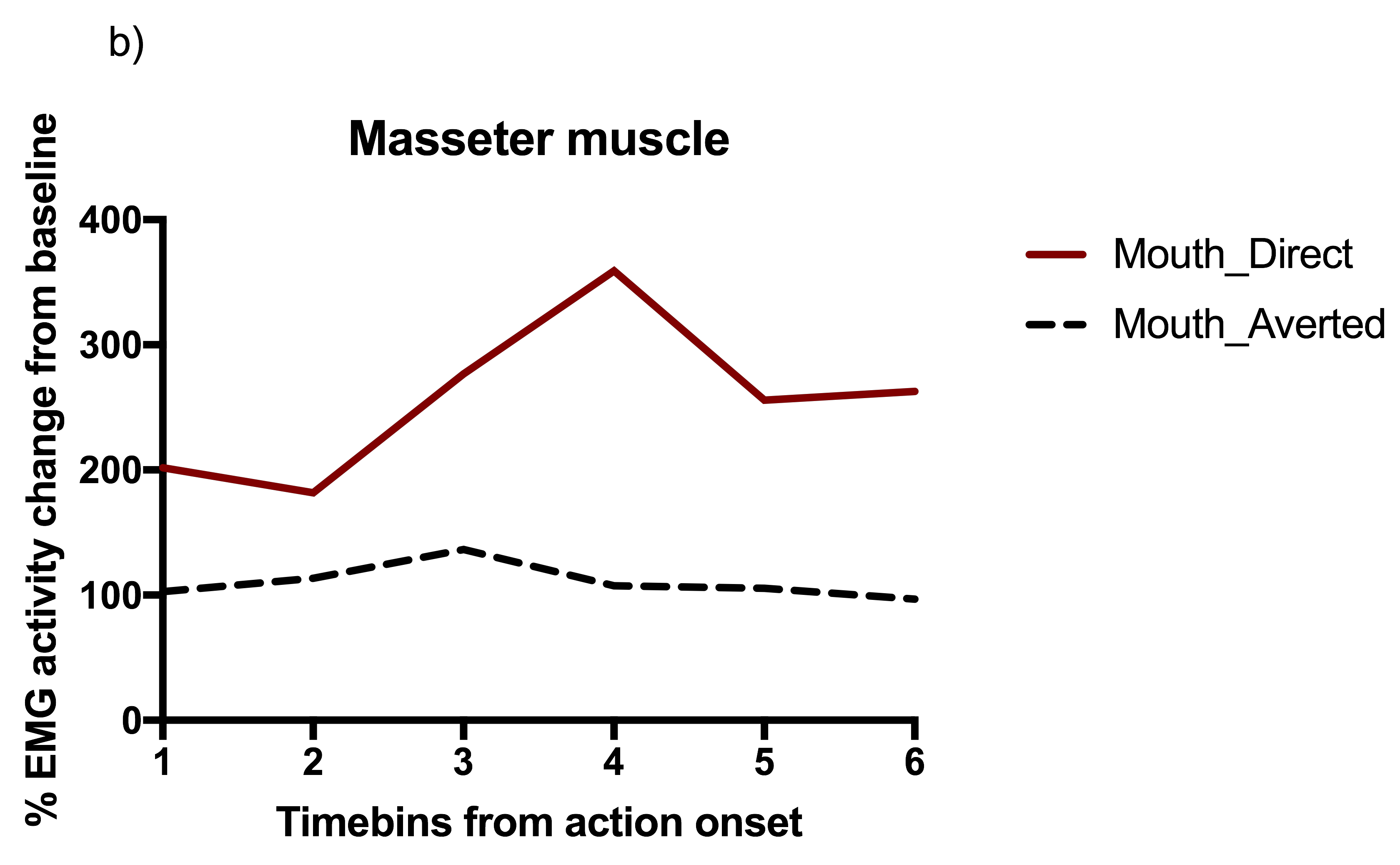
**

*Supplementary Figure 1.* Average EMG activity in percentage change from baseline over the frontalis (a) and masseter (b) muscle for each 500 ms timebin after action onset for one of the infants who showed a mimicry response in the face direct condition.

*Supplementary Table 1.* Channels that showed a significant hemodynamic response, i.e. an increase in HbO_2_ and/or a decrease in HHb during the observation of the facial action trials.

| **Channel** | **chromophore** | **F-value** | **p-value** |
| --- | --- | --- | --- |
| **Left Hemisphere** | | | |
| 4 | HbO_2_ | 3.774 | .007 |
| 4 | HHb | 2.803 | .030 |
| 6 | HbO_2_ | 3.556 | .009 |
| 7 | HbO_2_ | 13.257 | .000 |
| 8 | HbO_2_ | 3.949 | .005 |
| 9 | HbO_2_ | 2.432 | .051 |
| 10 | HbO_2_ | 4.764 | .001 |
| 12 | HbO_2_ | 6.264 | .000 |
| **Right Hemisphere** | | | |
| 14 | HbO_2_ | 3.565 | .009 |
| 18 | HbO_2_ | 4.852 | .001 |
| 20 | HbO_2_ | 9.456 | .000 |
| 21 | HHb | 2.885 | .026 |
| 22 | HbO_2_ | 3.288 | .014 |

**Eye-tracking data**

We recorded the looking behaviour during the observation of our stimuli from a subset of 14 infants using a Tobii TX300 eye tracker to investigate potential differences in overt attention to the facial actions accompanied by direct versus averted gaze. We defined rectangular areas of interest (AOIs) around the face, and calculated the proportion of time the infants spent looking at this face AOI relative to their total looking time towards the screen. Only trials with at least 2,000 ms of accumulated gaze data over the duration of the 10,000 ms video were included in the analyses. Infants were included in the analyses if they had at least 2 valid Direct gaze and 2 valid Averted gaze trials. We found that the proportion of time the infants spent looking at the face was significantly greater in the averted gaze compared to the direct gaze condition, *t*(13)=-2.242, *p*=.043 (Direct gaze M=0.886, Averted gaze M=0.912) . This suggests that the absence of mimicry in the averted gaze condition was unlikely the result of a lack of overt attention to the facial actions.

**Supplementary EMG analyses**

***Individual muscle activations***

In the paper we reported analyses performed on the Mimicry scores. Here we demonstrate that the results are identical when we analyse the individual muscle activations instead. A repeated measures analysis with Gaze direction (Direct vs. Averted), Muscle region (Frontalis vs. Masseter), and Action type (Eyebrow vs. Mouth) as within subject factors demonstrated a significant main effect of Muscle, *F* (1, 27) = 4.690, *p* = .039, *η_p_^2^* = .148, and a significant interaction between Gaze direction, Muscle region, and Action, *F* (1, 27) = 7.997, *p* = .009, *η_p_^2^* =.229. As can be seen in Supplementary Figure 2, the main effect was driven by stronger responses being present over the frontalis region compared to the masseter region. We followed-up on the significant three-way interaction by performing separate repeated measures analyses for the Direct and Averted gaze condition with Muscle region (Frontalis vs. Masseter), and Action type (Eyebrow vs. Mouth) as within subject factors. These analyses demonstrated a marginally significant interaction between Muscle and Action type in both conditions, Direct gaze, *F* (1, 27) = 3.382, *p* = .077, *η_p_^2^* =.111, and Averted gaze, *F* (1, 27) = 4.170, *p* = .051, *η_p_^2^* =.134. However, only in the Direct gaze condition was this interaction driven by more EMG activity being present over the corresponding muscle regions. Crucially, in this condition, there was significantly more frontalis region activation than masseter region activation during the observation of eyebrow actions, *t*(27)=2.085, *p*=.047, and significantly more frontalis region activation during the observation of eyebrow actions than during the observation of mouth actions, *t*(27)=2.108, *p*=.044 (See Supplementary Figure 2a). Additionally, only in the Direct gaze condition was the EMG activity over the frontalis region during the observation of eyebrow actions significantly different from zero, *t*(27)=2.409, *p*=.023. Thus, only in the Direct gaze condition was there evidence for mimicry, in particular over the eyebrow region.


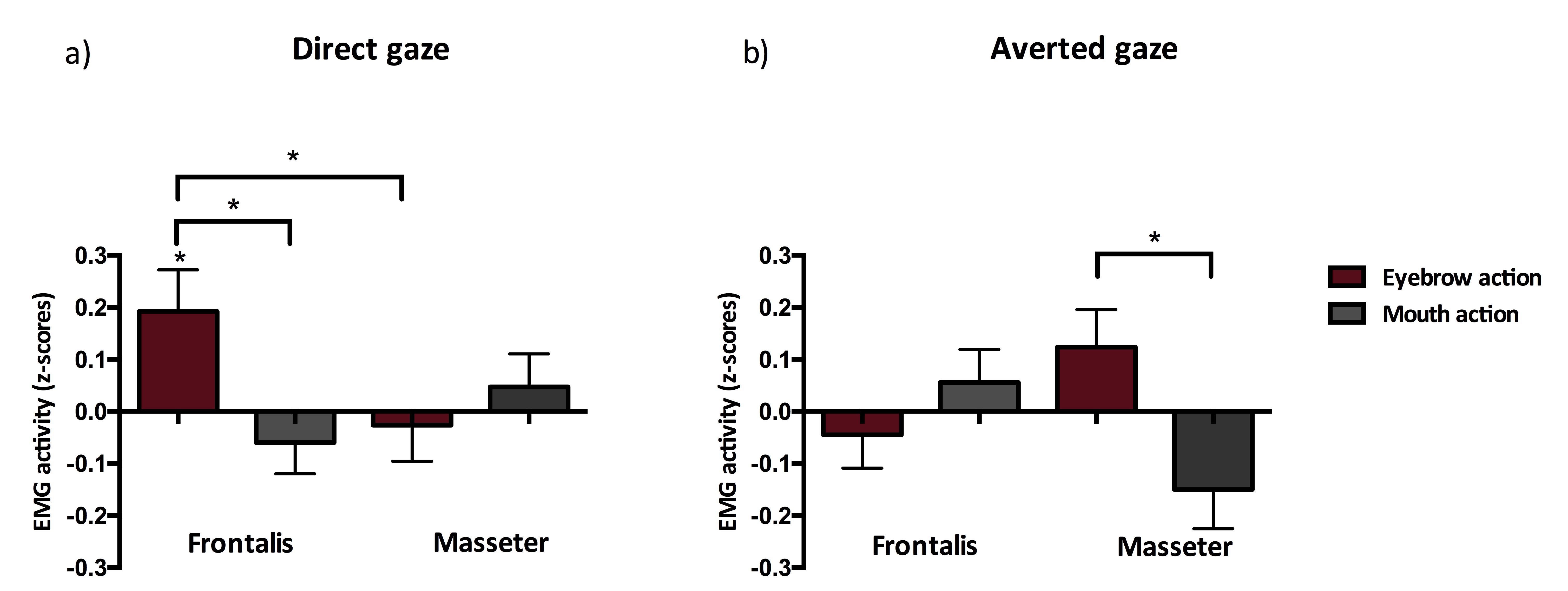


*Supplementary Figure 2.* a) Mean EMG-activity (z-scores) over the frontalis region and masseter region during the observation of eyebrow and mouth actions accompanied by direct gaze. * *p* < .05. Error bars indicate 1 SEM. b) Mean EMG-activity (z-scores) over the frontalis region and masseter region during the observation of eyebrow and mouth actions accompanied by averted gaze. * *p* < .05. Error bars indicate 1 SEM.

**Supplementary fNIRS analyses**

As we did not find evidence for mimicry of hand actions in Experiment 1, Experiment 2 focussed on the neural responses during the observation of facial actions accompanied by direct and averted gaze. Here we report the equivalent analyses for the neural responses during the observation of the hand action trials. As the hand action trials occurred less frequently than the facial action, infants with at least 2 trials per experimental condition (Hand_Direct, Hand_Averted) were included in the analyses. All the same infants who were included in Experiment 2 in the main text were also included in these analyses. On average, the included infants contributed 3.2 trials per condition to the analyses; 3.3 trials in the Hand_Direct condition, and 3.2 in the Hand_Averted condition. The number of included trials did not significantly differ between the Direct and Averted gaze condition, *p*=.63. The pre-processing steps and analysis approach were identical to those used for the analysis of the neural responses to the facial action trials.

The initial analyses identified 19 channels that showed a significant haemodynamic response, i.e. an increase in HbO_2_ and/or a decrease in HHb during the trial period compared to the baseline period (see Supplementary Table 2). For two of these channels we found a significantly greater HbO_2_ response to the Hand_Direct compared to the Hand_Averted condition (channel 2: main effect of condition, *F* (1,29) = 5.104, *p*=.032; indicating a greater HbO_2_ response to the Hand_Direct condition throughout the analysis period, and a significant interaction between time and condition *F*(4,116)=4.805, *p*=.001, indicating a significantly greater increase in the HbO_2_ response to the Hand_Direct condition over the analysis period; and channel 23: a significant interaction between time and condition *F*(4,116)=4.642, *p*=.002, indicating a significantly greater increase in the HbO_2_ response to the Hand_Direct condition over the analysis period). Using a standardized scalp surface map of the fNIRS channel coordinates for this array and this age range (Lloyd-Fox et al., 2014) we identified the location of these channels as overlying the left IFG (channel 2) and right STS region (channel 23). Although channels 2 and 23 exhibit a significant effect of condition at the single channel level, there were no significant effects on the neighbouring channels, and so under our two adjacent channel criterion, these effects are not interpreted as statistically important. None of the other channels showed an effect of condition. We did not find any relationships between activation over channel 2 or 23 and the Hand mimicry scores, all *p*’s > .205.

*Supplementary Table 2.* Channels that showed a significant hemodynamic response i.e. an increase in HbO_2_ and/or a decrease in HHb during the observation of the hand action trials.

| **Channel** | **chromophore** | **F-value** | **p-value** |
| --- | --- | --- | --- |
| **Left Hemisphere** | | | |
| 2 | HbO_2_ | 7.616 | .000 |
| 3 | HbO_2_ | 3.907 | .005 |
| 3 | HHb | 2.838 | .027 |
| 6 | HbO_2_ | 2.664 | .036 |
| 7 | HbO_2_ | 4.781 | .001 |
| 8 | HbO_2_ | 3.119 | .018 |
| 8 | HHb | 3.475 | .010 |
| 10 | HbO_2_ | 7.761 | .000 |
| 12 | HbO_2_ | 15.199 | .000 |
| 12 | HHb | 15.046 | .000 |
| 13 | HbO_2_ | 13.731 | .000 |
| 13 | HHb | 5.094 | .001 |
| **Right Hemisphere** | | | |
| 14 | HbO_2_ | 5.082 | .001 |
| 16 | HbO_2_ | 8.615 | .000 |
| 16 | HHb | 2.634 | .037 |
| 18 | HbO_2_ | 4.922 | .001 |
| 19 | HbO_2_ | 4.931 | .001 |
| 20 | HbO_2_ | 4.294 | .003 |
| 21 | HbO_2_ | 2.980 | .023 |
| 22 | HHb | 2.454 | .050 |
| 23 | HbO_2_ | 6.954 | .000 |
| 24 | HbO_2_ | 3.898 | .005 |
| 25 | HbO_2_ | 20.800 | .000 |
| 25 | HHb | 4.576 | .002 |
| 26 | HbO_2_ | 16.189 | .000 |
| 26 | HHb | 7.632 | .000 |
